# Cathode Materials for Photocatalytic Fuel Cells: Design Strategies, Reaction Mechanisms, and Wastewater Treatment Applications

**DOI:** 10.3390/nano16160995

**Published:** 2026-08-12

**Authors:** Xingshun Zhu, Fei Li, Qiyuan Chen, Yizhen Zhang

**Affiliations:** 1 College of Safety and Environmental Engineering, Shandong University of Science and Technology, Qingdao 266590, China; 202311020429@sdust.edu.cn; 2 Hohhot Energy Conservation and Carbon Reduction Center, Hohhot 010000, China; 3 Ability R&D Energy Research Centre, School of Energy and Environment, City University of Hong Kong, Hong Kong 999077, China

**Keywords:** cathode materials, oxygen reduction reaction, pollutant degradation, electricity generation

## Abstract

Photocatalytic fuel cells (PFCs) integrate photocatalysis with fuel cell technology to enable simultaneous wastewater treatment and energy recovery. This review examines recent advances in PFC cathode materials, focusing on design strategies, reduction mechanisms, and applications. The cathode governs electron transfer and interfacial reactions, including oxygen reduction (4e^−^ or 2e^−^ pathways), direct pollutant electroreduction, and oxidant activation for radical generation. Cathodic materials including transition metal oxides/sulfides, carbon-based materials, metal–organic frameworks and their derivatives, are systematically summarized, evaluating their respective activities, stabilities and costs. Rational design via heterojunction engineering, defect modulation, and composite construction enables tunable reaction pathways and enhanced performance. Furthermore, representative applications are reviewed, with particular attention to the effective degradation of organic pollutants, and reduction of heavy metals and radionuclides in PFCs. Future efforts should prioritize long-term stability, scalable fabrication, and multi-functional cathode integration.

## 1. Introduction

The global energy crisis and environmental pollution have driven the search for sustainable technologies that can simultaneously address both challenges [1]. Photocatalytic fuel cells (PFCs) have emerged as a promising dual-function platform that integrates photocatalysis with fuel cell technology, enabling simultaneous wastewater treatment and clean energy recovery using only solar energy [2,3]. A typical PFC comprises a photoanode that generates electron–hole pairs under light irradiation and a cathode where reduction reactions occur [4]. This configuration allows organic pollutants to be oxidized by photogenerated holes at the photoanode, while electrons are transferred to the cathode via an external circuit to produce electricity [3].

The cathode is critical to PFC performance, as it governs electron transfer and drives interfacial reduction reactions [1]. Key cathodic processes include the oxygen reduction reaction (ORR), which can proceed via a four-electron pathway to yield water, or a two-electron route producing hydrogen peroxide [2]. The cathode can also directly reduce high-valence toxic pollutants such as Cr (VI) and U (VI) into less harmful forms [3], and activate exogenous oxidants like peroxymonosulfate to generate reactive radicals that significantly enhance oxidative degradation [5]. Moreover, the proton reduction reaction at the cathode (2H^+^ + 2e^−^ → H_2_) under anaerobic conditions provides a sustainable route for hydrogen generation, aligning PFCs with the broader electrochemical green-hydrogen framework [6,7,8]. The rational design of cathode materials—balancing conductivity, active sites, and chemical robustness—is therefore essential for optimizing PFC performance [9].

In photocatalytic fuel cells, the choice of cathode material cannot be based on a single criterion or a general-purpose solution. Instead, it necessitates a systematic optimization that reconciles competing factors, namely catalytic performance, long-term stability, economic viability, and scalable production processes. In practice, transition-metal oxides and sulfides—thanks to their readily tunable electronic structures and abundant redox chemistry—remain the workhorses of the field. Recently, the carbon cathodes investigated in PFC studies include NC/CF [10], CCNTs/PPy/GF [11], and ZnS/CF [12]. Carbonaceous supports such as felts, graphene hybrids, and conductive-polymer composites complement them with superior charge-transfer kinetics and exceptional corrosion resistance, making this oxide-carbon pairing the most commercially viable backbone for large-scale deployments. Han et al. [13] investigated a Cu_2_O/Cu oxide cathode in a photocatalytic fuel cell for simultaneous organic pollutant degradation and electricity generation, whereas Nahyoon’s group [14] studied a ZnIn_2_S_4_ photocathode, which is a sulfide material rather than an oxide. Additionally, metal–organic frameworks (MOFs) and their pyrolyzed derivatives, for instance, bring molecular-scale precision and exceptionally high surface areas to the catalytic interface, though their long-term stability often demands careful engineering [15]. Noble metals, despite their benchmark catalytic prowess, are generally relegated to auxiliary modifiers owing to prohibitive costs [1,16], whereas perovskite-type oxides carve out a niche in aggressive or high-temperature environments by leveraging their extraordinary oxygen mobility and structural stability [17]. Meanwhile, intrinsically conducting polymers add yet another dimension—their mechanical flexibility and doping-tunable conductivity render them especially attractive for next-generation, lightweight, or even wearable PFC architectures [2]. Ultimately, no single material excels in every respect; the optimal choice hinges on the specific operating conditions and economic constraints [2,17], and often a synergistic combination of these diverse chemistries proves more promising than any individual contender [3,15].

Several comprehensive reviews have explored PFC development from different perspectives. Li et al. [18] systematically reviewed the fundamentals, working principles, and configuration development of PFCs, with particular emphasis on reactor configurations and coupled advanced oxidation processes for enhancing wastewater treatment performance. A study by Ni et al. [7] presented a comprehensive assessment of the current status, challenges, and perspectives of PFCs, covering photoanode/photocathode materials, cathode materials, system configurations, and radical reaction processes. Mishra’s group [19] offered a nuanced review of the critical challenges facing photocatalytic fuel cells in sustainable power production, addressing catalyst stability, charge separation efficiency, and the practical barriers to large-scale implementation. However, none of these reviews have specifically concentrated on the systematic screening, construction, and application mechanisms of cathode materials in PFCs for environmental remediation.

This review first provides a systematic overview of the fundamental reduction mechanisms at PFC cathodes, encompassing the ORR, direct electroreduction of pollutants, and cathodic activation of exogenous oxidants. It then presents a comprehensive classification and comparative analysis of state-of-the-art cathode materials—covering transition metal oxides/sulfides, carbon-based materials, MOFs and their derivatives, and other emerging candidates—with emphasis on design strategies and structure–activity relationships. Finally, the application performance of these cathodes in wastewater treatment, heavy metal/radionuclide removal, and simultaneous electricity generation is critically evaluated, followed by a discussion of current challenges and future research directions. By integrating mechanistic insights with material engineering and application-oriented assessment, this review aims to provide strategic guidance for the rational development of high-performance PFC cathodes and to promote the advancement of sustainable water–energy technologies.

## 2. Fundamentals of PFCs

### 2.1. Working Principle and Charge-Carrier Dynamics

**Figure 1:**
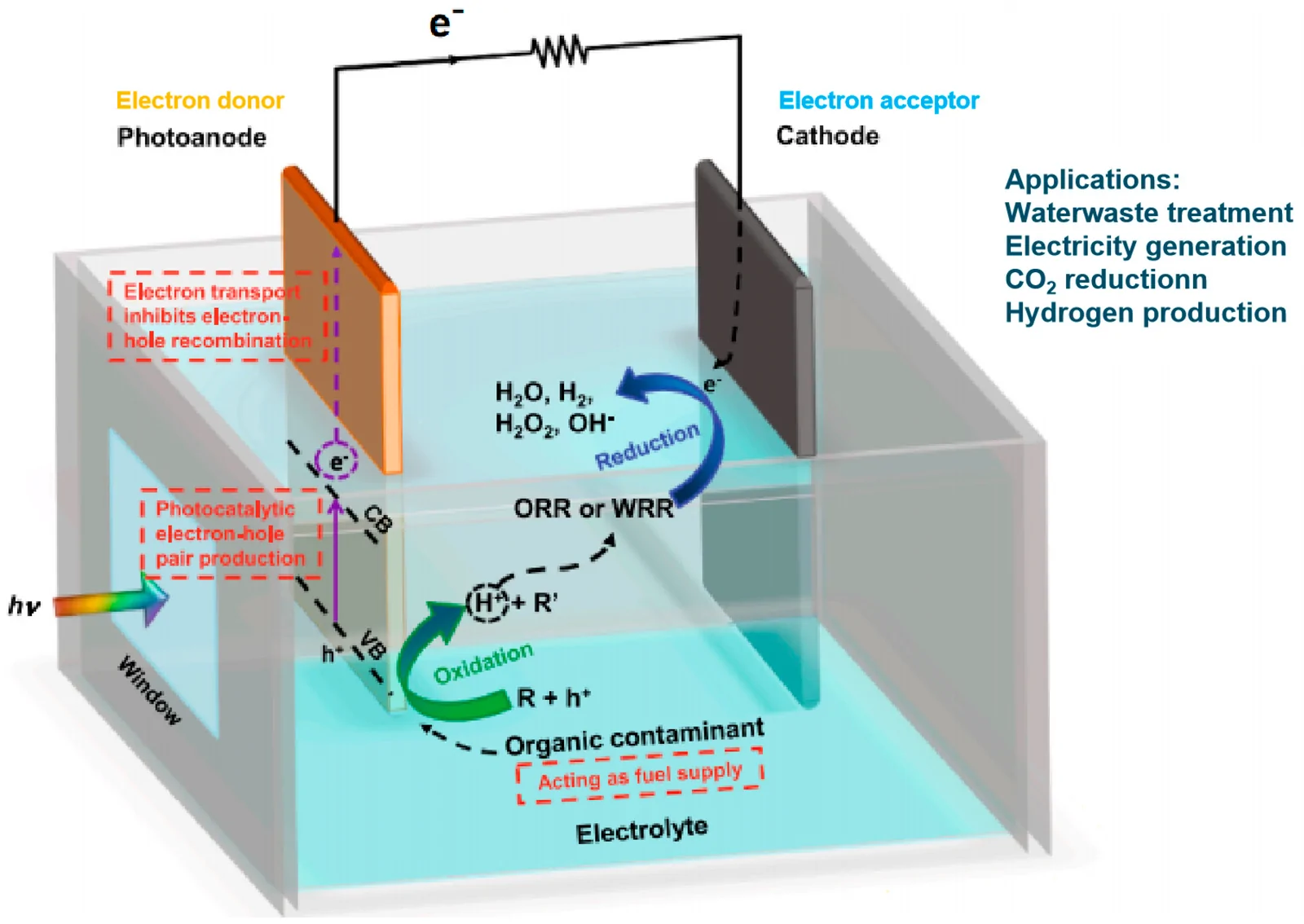
Schematic diagrams of integration to PFC [23].

A PFC integrates photocatalysis with fuel cell technology to achieve simultaneous wastewater treatment and electricity generation [20]. As depicted in Figure 1, illumination of the photoanode generates electron–hole pairs; photogenerated holes oxidize organic contaminants, while electrons migrate through the external circuit to the cathode, driven by the inter-electrode Fermi-level gradient [9,20]. This directional transport generates current and suppresses recombination. The cathode constitutes the functional core, governing electron consumption and interfacial reduction reactions [7]. Its dual photocatalytic/electrocatalytic role is critical: electrocatalytically, it drives the oxygen reduction reaction (ORR); photocatalytically, p-type semiconductor cathodes in dual-photoelectrode configurations create an internal bias via Fermi-level mismatch, enhancing charge separation [9,21]. Additionally, cathodic materials can activate peroxymonosulfate to generate sulfate radicals (SO_4_•^−^), extending oxidative degradation [22]. Overall performance depends on the synergy among thermodynamic feasibility, kinetic accessibility, and long-term stability [7].

Rational design of integrated dual-photoelectrode systems requires careful band alignment [7,9]. Four principles govern this: (i) the Fermi-level gradient drives spontaneous electron migration; (ii) conduction-band offsets must ensure photoanode electrons reach the photocathode while preserving strongly reducing electrons for target redox reactions [24]; (iii) the cathode surface must provide favorable kinetics and sufficient overpotential for the target reduction reaction to outcompete dissolved oxygen and other parasitic electron acceptors [25]; and (iv) the cathode bandgap and band-edge positions must simultaneously satisfy visible-light harvesting and the thermodynamic requirements of target reductions [3,14,24,26]. Notably, dissolved oxygen competes with pollutants for photoelectrons, often enhancing power output at the expense of reductive treatment efficiency [27].

### 2.2. Cathodic Reaction Pathways and Underlying Mechanisms

#### 2.2.1. Oxygen Reduction Reaction Pathways

**Figure 2:**
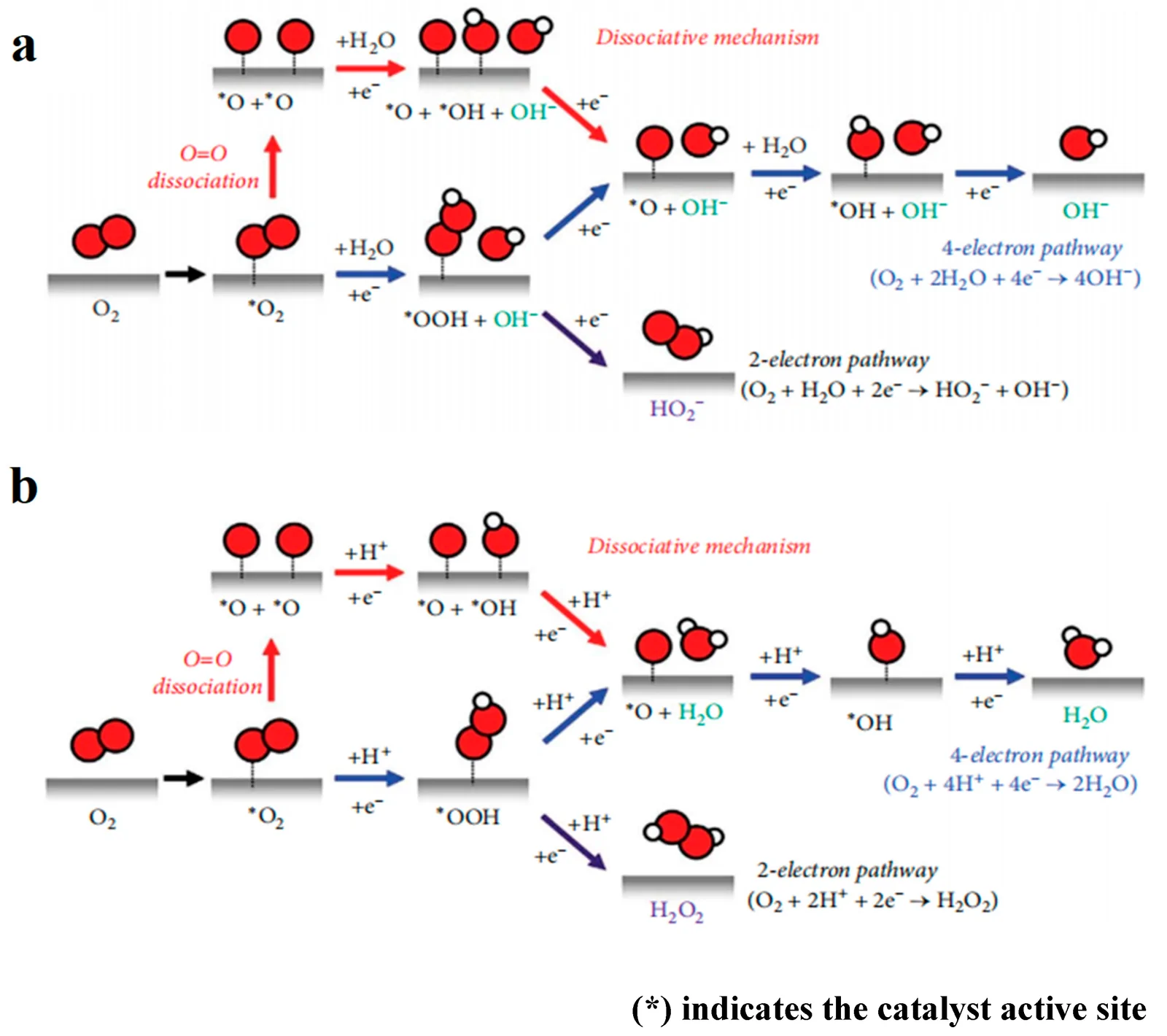
Two schematic diagrams illustrating the proposed ORR mechanisms under alkaline (**a**) and acidic (**b**) conditions, respectively [28].

The ORR is the dominant electron-accepting process at the cathode under aerobic conditions. Two pathways dictate distinct application outcomes (Figure 2). The four-electron pathway (O_2_ + 4H^+^ + 4e^−^ → 2H_2_O, E^0^ = +1.23 V vs. NHE) maximizes energy recovery by extracting the full chemical potential of O_2_ and is kinetically favored on materials with strong O_2_ adsorption and low O–O dissociation barriers [28]. Conversely, the two-electron pathway (O_2_ + 2H^+^ + 2e^−^ → H_2_O_2_, E^0^ = +0.68 V vs. NHE) preserves the O–O bond, yielding H_2_O_2_ as a soluble product that can be activated to •OH for advanced oxidation [28,29]. Pathway selectivity is governed by the electronic structure and active-site geometry of the cathode: high active-site density and upward-shifted d-band centers favor 4e^−^ selectivity, whereas site isolation, heteroatom doping, or moderate *OOH binding energies promote 2e^−^ selectivity [30,31].

#### 2.2.2. Direct Electroreduction of Pollutants

Photogenerated electrons with sufficiently negative reduction potentials can directly reduce high-valence pollutants without external chemical reductants. This pathway is thermodynamically feasible when the cathode conduction-band edge lies above the pollutant’s reduction potential. It is particularly effective for redox-active heavy metals (e.g., Cr (VI) → Cr (III)) and radionuclides (e.g., U (VI) → U (IV)), where the reduction products are less toxic or more readily separable [3,26,32]. In dual-photoelectrode configurations, inter-electrode charge transfer preserves the most reducing electrons at the cathode while consuming holes at the photoanode, minimizing recombination losses [24]. Post-reaction surface characterization is typically required to distinguish true reduction from adsorption-dominated removal [3].

#### 2.2.3. Cathodic Activation of Exogenous Oxidants

**Figure 3:**
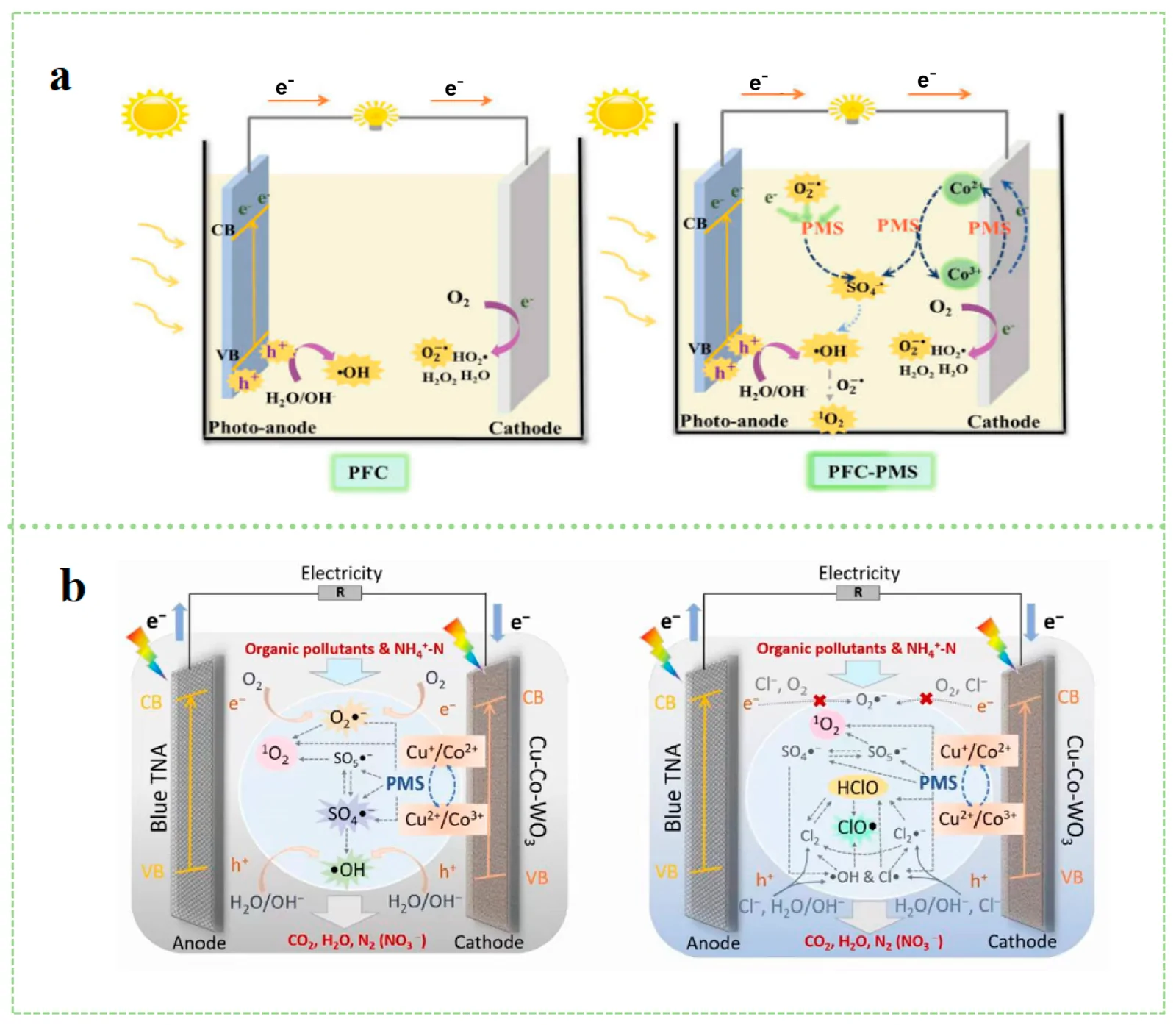
Reaction mechanisms of PFC-based systems. (**a**) A schematic diagram illustrating the reaction mechanism of the PFC/PFC-PMS system under visible-light irradiation, which employs an Ag/ZnO photoanode and a CoFe_2_O_4_/CC cathode for simultaneous wastewater treatment and electricity generation [33]; (**b**) a proposed mechanism of the PFC/PMS system in two distinct aquatic environments: freshwater (**left**) and marine (**right**) [18].

Beyond ORR and direct reduction, cathode-accumulated electrons can activate externally supplemented oxidants such as peroxymonosulfate (HSO_5_^−^) and persulfate (S_2_O_8_^2−^). This reductive activation cleaves the peroxide O–O bond via one-electron transfer, generating sulfate radicals (SO_4_•^−^, E^0^ = 2.5–3.1 V vs. NHE), which may further react with water or hydroxide to yield •OH [33]. The process is synergistically enhanced when cathode surfaces possess redox-active metal couples (e.g., Co^3+^/Co^2+^, Cu^2+^/Cu^+^) that cycle oxidant activation while photoelectrons sustain the catalytic loop [19]. In high-salt matrices, electrolyte ions such as Cl^−^ can intercept primary radicals and redirect the reactive-species landscape toward reactive chlorine species, fundamentally reconstructing oxidation pathways without altering the cathode material itself [18,22] (Figure 3).

#### 2.2.4. Synergistic Multi-Mechanism Coupling

High-performance cathodes rarely rely on a single reaction pathway; instead, they couple multiple mechanisms to achieve synergistic outcomes. Functional complementarity enables simultaneous removal of disparate pollutants—for example, reductive immobilization of heavy metals alongside oxidative mineralization of organics. Reaction-pathway coupling occurs when the product of one cathodic process (e.g., H_2_O_2_ from 2e^−^ ORR) serves as the reactant for a subsequent step (e.g., Fenton oxidation), creating an auto-amplifying degradation cycle on the same interface [20,34]. Such synergy demands cathode materials that integrate multiple active domains: sites for electron transfer to pollutants, sites for ORR with tunable selectivity, and sites for oxidant activation.

### 2.3. Influence of Operational Parameters on Cathode Performance and Reaction Selectivity

The cathodic reaction pathway is critically governed by the interfacial microenvironment established by pH, dissolved oxygen (DO), and electrolyte composition [20,22]. These variables collectively modulate thermodynamic driving forces and interfacial charge-transfer dynamics, determining the competition among oxygen reduction, direct pollutant electroreduction, and exogenous oxidant activation [34]. Specifically, pH regulates ORR mechanism equilibrium—alkaline conditions favor the dissociative 4e^−^ pathway, whereas acidic media promote the associative 2e^−^ route—while simultaneously governing proton-coupled electron transfer kinetics for direct reduction in high-valence pollutants [28,32]. Dissolved oxygen serves as the primary electron acceptor under aerobic conditions, yet its concentration critically determines the allocation of photogenerated electrons between energy recovery and reductive pollutant treatment, establishing a competitive dynamic that dictates overall system selectivity [27]. Furthermore, electrolyte composition introduces ion-specific effects; halide ions in complex matrices can rapidly scavenge primary radicals and redirect oxidation mechanisms toward alternative reactive species, thereby reconstructing degradation pathways [22]. Consequently, these operational parameters form an interconnected design space, and rational engineering of PFC systems necessitates the simultaneous optimization of material properties and operational conditions to achieve tunable reaction selectivity across diverse wastewater chemistries.

## 3. Classification of Cathode Materials

### 3.1. Transition Metal Oxides and Sulfides

Transition metal oxides and sulfides stand out as the most extensively investigated noble-metal-free PFC cathode candidates, owing to their unique ability to synergistically combine intrinsic high ORR activity with highly tunable band structures that enable efficient visible-light excitation, thereby significantly reducing the ORR overpotential through the modulation of photogenerated charge carriers [1,35,36]. Relative to Pt-based cathodes, they offer orders-of-magnitude lower cost and easily tailorable electronic properties via doping and heterostructure engineering [30]. In comparison with carbon-based cathodes, their semiconducting character intrinsically couples photo response and catalysis, introducing extra degrees of freedom for performance optimization [37,38]. Mechanistically, their sufficiently negative conduction band minima facilitate both 2e^−^ and 4e^−^ ORR pathways, while their valence bands can be engineered for enhanced visible-light absorption [39]. Furthermore, the presence of variable-valence metal sites serves a dual role—acting as ORR active centers and redox mediators for external oxidant activation. Consequently, substantial research efforts have been directed toward leveraging these materials for high-performance PFC cathodes [21].

#### 3.1.1. Transition Metal Oxides

Transition metal oxides have been extensively investigated as PFC cathode materials [1,30,36,37,40,41,42,43], owing to their low cost, structural diversity, and tunable electronic properties; a large number of studies have further focused on overcoming their intrinsic limitations, such as poor conductivity and photocorrosion, through various modification strategies.

Cuprous oxide (Cu_2_O) is a typical p-type semiconductor with a band gap of 2.0–2.3 eV and a conduction band edge as negative as −1.4 V vs. NHE [36,42]. Thanks to its superior visible-light response and inherent ORR activity, it ranks among the earliest oxide materials explored for PFC cathodes. However, its practical application is limited by poor intrinsic conductivity (~10^−4^–10^−3^ S cm^−1^) and severe photocorrosion, where photogenerated holes oxidize Cu^+^ to Cu^2+^ [30]. Accordingly, state-of-the-art modification strategies are designed to target two core issues: accelerating carrier separation and suppressing hole accumulation at Cu^+^ sites. A widely validated strategy is the construction of metal oxides–semiconductor heterojunctions integrated with conductive carbon supports. A study by Li et al. [30] fabricated Cu–Cu_2_O nanoparticles supported on electrospun carbon nanofibers. High-Resolution Transmission Electron Microscopy (HRTEM) images revealed distinct lattice fringes of metallic Cu and Cu_2_O, confirming a tightly contacted heterointerface. The resulting built-in electric field drives spatial charge separation, directing electrons to Cu sites and holes to the CNF skeleton, which simultaneously mitigates photocorrosion and promotes charge separation. This structure reduces the ORR rate-determining step energy barrier from 1.31 eV (pure Cu_2_O) to 1.27 eV. The assembled PFC, paired with a WO_3_/rGO photoanode, achieved 83% RhB degradation in 2 h and a maximum power density of 42.99 μWcm^−2^.

Manganese dioxide (MnO_2_) is an eco-friendly, low-cost transition metal oxide whose surface Mn^3+^/Mn^4+^ redox couple provides intrinsic ORR activity [37]. Layered δ-MnO_2_ possesses a large interlayer spacing that facilitates O_2_ adsorption and electrolyte ion diffusion. However, its intrinsic conductivity is only 10^−5^–10^−6^ S cm^−1^. Composite hybridization with conductive carbon materials represents an efficient solution to this bottleneck. Ouyang et al. [37] synthesized a MnO_2_/graphene oxide (GO) composite cathode via a hydrothermal method. Composite hybridization with conductive carbon materials represents an efficient solution to this bottleneck. Paired with a BiVO_4_/WO_3_ inverse opal photoanode, the cathode delivered a power density of 66.2 μWcm^−2^ and a short-circuit current density of 593.5 μAcm^−2^, 2.2- and 2.3-fold higher than pure MnO_2_.

Extending beyond single-phase oxides, Bi-based oxyhalides (e.g., BiOBr, BiOCl) possess a unique layered structure with an internal electric field that promotes interlayer carrier separation. Their band structures can be tuned via halogen regulation and solid-solution formation, making them ideal for constructing S-scheme heterojunctions [25,44]. S-scheme heterojunctions selectively recombine carriers with weak redox potentials while retaining strong ones, perfectly balancing carrier separation and redox capacity for PFC cathodes. In one study, Yu and co-workers [25] fabricated an S-scheme BiOAc_1−x_Br_x_/BiOBr photocathode. HRTEM images showed lattice fringes of 0.285 nm (BiOBr) and 0.279 nm (BiOAc_1−x_Br_x_) in intimate contact. Ultraviolet Photoelectron Spectroscopy (UPS) and Density Functional Theory (DFT) calculations confirmed the S-scheme charge transfer pathway, which preserves strongly reducing CB electrons and significantly enhances ORR kinetics. The PFC system achieved 96.33% tetracycline degradation and a maximum power density of 714 μWcm^−2^.

#### 3.1.2. Metal Sulfides

Compared with metal oxides, metal sulfides exhibit distinct electronic advantages: their valence bands originate from the hybridization of S 3p and metal orbitals, endowing them with more negative VB positions and narrower band gaps (typically 1.8–2.4 eV) [39], which further expand the visible-light harvesting range. Notably, ternary sulfides generally outperform binary sulfides in terms of structural stability and resistance to photocorrosion [14].

As a prototypical example, zinc indium sulfide (ZnIn_2_S_4_) is a representative ternary layered sulfide with a band gap of ~2.1 eV and a conduction band edge as negative as −1.1 V vs. NHE, integrating exceptional visible-light response, efficient carrier transport, and satisfactory ORR activity. Nahyoon et al. [14] employed ZnIn_2_S_4_ as a photocathode coupled with an Ag_3_PO_4_/Fe/GTiP photoanode. The system achieved 90% RhB degradation within 70 min under visible light, an open-circuit voltage of 0.18 V, while maintaining stable performance over four consecutive cycles, fully demonstrating the promising potential of ZnIn_2_S_4_ as a noble-metal-free PFC cathode. In a different study, Wang and colleagues [39] employed a one-step hydrothermal method to grow SnS_2_ nanoplates on the surface of carbon felt (CF) fibers.

**Figure 4:**
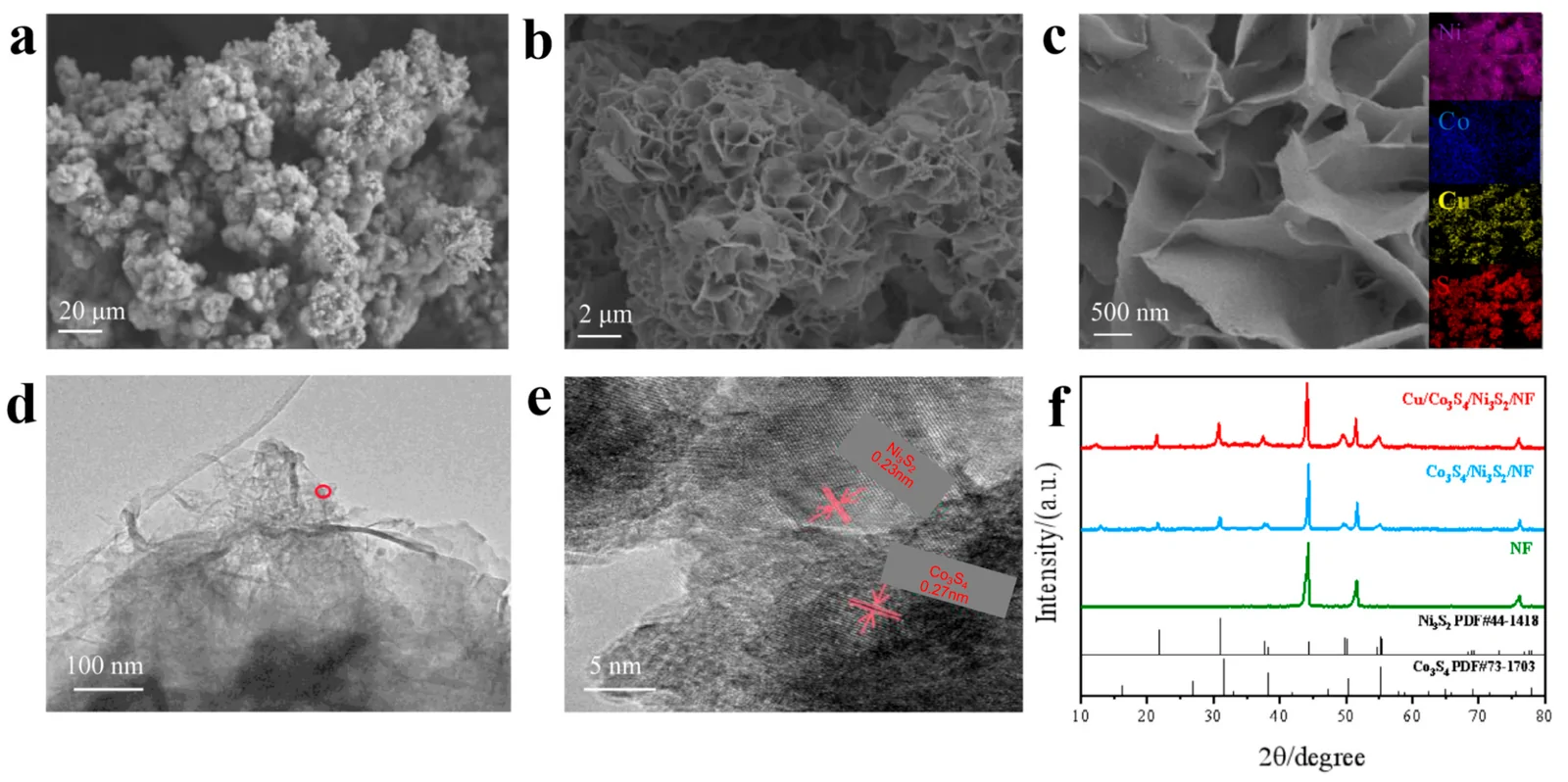
(**a**–**c**) SEM images and (**d**,**e**) TEM images of Cu/Co_3_S_4_/Ni_3_S_2_; (**f**) XRD patterns of prepared cathodes [45].

To further enhance charge transfer kinetics, heterostructured bimetallic sulfides have been developed. Wang et al. [45] reported a Cu-doped Co_3_S_4_/Ni_3_S_2_ cathode featuring a flower-ball morphology assembled from ultrathin nanosheets (Figure 4a–c). EDS mapping confirms the uniform distribution of Ni, Co, Cu, and S. TEM images (Figure 4d,e) reveal lattice fringes of 0.27 nm and 0.23 nm, corresponding to the plane of Co_3_S_4_ and the (021) plane of Ni_3_S_2_, respectively. XRD patterns (Figure 4f) verify the coexistence of Ni_3_S_2_ and Co_3_S_4_ phases, with a negative peak shift indicating Cu incorporation into the lattice. When integrated with a CuO QDs/TiO_2_/WO_3_ photoanode, this cathode achieved 93.4% rifampicin removal and a maximum power density of 500 μW cm^−2^, demonstrating the effectiveness of bimetallic sulfide heterojunctions.

Overall, metal oxide/sulfide cathodes leverage p-type semiconducting properties, visible-light activity, and compatibility with advanced oxidation processes. Key subclasses—Cu-based oxides, Fe-based spinels, metal sulfides, Mn oxides, and Bi-based oxyhalides—continuously improve PFC performance through enhanced charge utilization and radical generation. Future research priorities should center on nanostructure design, defect engineering, and heterojunction integration, aiming to further improve conductivity, stability, and selectivity for practical wastewater treatment applications.

### 3.2. Carbon-Based Materials

In recent years, carbon-based cathode materials have attracted extensive research attention owing to their low cost [45], excellent electrical conductivity [46,47], good chemical stability [48], and highly tunable structures [49,50], and are widely considered one of the most promising Pt-free alternatives for photocathodes in PFC [10,11]. Considerable efforts have been devoted to developing high-performance carbon-based cathodes [12,30], whose core function is to efficiently accept photogenerated electrons [31] and catalyze the ORR [42], a process that directly determines the charge separation efficiency and power output performance of PFCs [51,52,53]. Based on differences in material composition and structural design, current research on carbon-based cathodes can be broadly divided into two main categories: single-component carbon cathodes and carbon-based composite or hybrid cathodes.

#### 3.2.1. Single-Component Carbon Cathodes

Carbon-based materials are promising Pt-free cathodes for PFCs owing to their low electrical impedance, large specific surface area, good chemical stability, and structural tunability [50,53]. In studies on single carbon structures, i.e., pure carbon forms without metal oxides, polymers, or other carbon allotropes, the focus is placed on regulating the macroscopic morphology (fibers, felts, cloths, plates, particles, etc.) and pore architecture. Such structural modulation optimizes the ORR mass transfer process and exposes more catalytically active sites, enabling an accurate evaluation of the intrinsic catalytic performance of pristine carbon substrates [49].

Three-dimensional (3D) porous carbon fiber-based matrices are the most common configuration for single-carbon cathodes. Thor et al. [50] systematically compared carbon felt, carbon plate, and carbon cloth in a PFC-electro-Fenton integrated system. The unique 3D interconnected fibrous network of CF greatly facilitates oxygen adsorption and diffusion, achieving an electrogenerated H_2_O_2_ concentration of 0.289 mM, which is remarkably higher than that of CP (0.107 mM) and CC (0.043 mM). Benefiting from the superior ORR mass transfer capability, the CF cathode achieved 97.47% decolorization of Amaranth dye in PFC mode, with a maximum power density (P_max_) of 3.560 μW cm^−2^, which was 2.5- and 12.1-fold higher than those of CP and CC, respectively. This study verifies that macroscopic morphological differences among pure carbon materials can induce performance discrepancies by orders of magnitude, confirming the decisive role of 3D porous structures in accelerating ORR kinetics and improving PFC power output.

Macroscopic electrode configuration engineering is another effective strategy to boost mass transfer efficiency. Zhang’s group [53] designed a rotating multi-cathode system consisting of four parallel bamboo charcoal (BC)-loaded titanium (Ti) plates, aiming to alleviate the mass transfer limitation under high catalyst loading conditions. Under a fixed total BC loading of 8 mg cm^−2^, the multi-cathode system exhibited an electrochemical active area 3.6 times larger than that of a conventional single cathode, along with a sharply reduced electron transfer resistance (R_et_) of 1.01 Ω, in contrast to 9.38 Ω for the single cathode. Correspondingly, the P_max_ of the multi-cathode system reached 0.22 mW, which was 53% higher than that of the single cathode and even superior to the commercial Pt cathode. This work demonstrates that low-cost biomass-derived carbon (bamboo charcoal) can achieve performance close to noble metals through rational flow-field and electrode arrangement, offering a practical path for scaling up single-carbon cathodes.

Chemical activation provides an additional route to enhance the ORR activity of single-carbon cathodes. Thor et al. [54] treated carbon felt with KOH. Meanwhile, a study by Wang [55] reveals a significantly higher oxygen distribution on the activated carbon felt, confirming the introduction of oxygen-containing functional groups. These modifications improved the hydrophilicity and porosity of the cathode, thereby boosting H_2_O_2_ electro-generation by more than threefold. As a result, the activated cathode achieved >99% Amaranth decolorization in the PFC-PEF system and delivered a 22% higher power density than pristine carbon felt, with stable performance maintained over five cycles [41].

#### 3.2.2. Carbon-Based Composite and Hybrid Cathodes

Carbon-based composite cathodes, fabricated by integrating carbon materials with conductive polymers, metal oxides, or other carbon allotropes, can generate prominent synergistic effects and achieve comprehensive performance improvement.

A PPy/MWCNTs/Fe_3_O_4_ composite cathode was constructed by electrodepositing the nanocomposite onto a graphite substrate [29]. Structural characterization reveals that MWCNTs are uniformly embedded in the PPy matrix, forming a continuous porous conductive network, while Fe_3_O_4_ nanoparticles (80–100 nm) are evenly dispersed on the composite surface. This architecture not only provides abundant active sites and enhanced electrical conductivity for efficient oxygen reduction, but also enables the in situ electrogeneration of H_2_O_2_ via the 2e^−^ ORR pathway (with a maximum production of 11.5 mg L^−1^ at −0.55 V vs. SCE), which is subsequently activated by the LDH/PS system to generate sulfate radicals (SO_4_•^−^) and hydroxyl radicals (•OH) for enhanced pollutant degradation. Graphene-based composites have emerged as a prominent class of cathode materials owing to their exceptional electrical conductivity and structural tunability. Likewise, MnO/GO cathodes deliver substantially higher power densities than bare MnO_2_ [37].

Performance optimization of carbon-based cathodes generally follows three strategic avenues: (1) constructing 3D porous structures to enhance mass transport (e.g., carbon felt, multi-cathode systems) [50,53]; (2) compositing with conductive polymers, graphene, or metal oxides to synergistically improve conductivity and catalytic activity [29,37,51]; and (3) utilizing low-cost carbon sources (e.g., biochar, candle soot) to achieve economic sustainability [48]. Beyond these general strategies, the rational tuning of ORR selectivity represents a critical design consideration that directly determines the application direction of carbon-based cathodes. Pristine carbon materials (e.g., carbon felt, graphene) typically favor the 2e^−^ ORR pathway, as their sp^2^ carbon edges and defective sites bind *OOH moderately, promoting its release as H_2_O_2_ rather than further reduction to H_2_O. This intrinsic property makes undoped or mildly functionalized carbons ideal platforms for PFC–electro-Fenton integration, where electrogenerated H_2_O_2_ serves as the key reagent for pollutant degradation. Conversely, introducing nitrogen dopants (e.g., pyridinic-N, graphitic-N) or single-atom metal–nitrogen (M–N_x_) coordination sites strengthens O_2_ adsorption and lowers the barrier for O–O dissociation, effectively switching the pathway toward 4e^−^ and enhancing power output.

### 3.3. Metal–Organic Frameworks (MOFs) and Derivatives

Metal–organic frameworks have emerged as a versatile platform for PFC cathode design, owing to their crystalline porous architecture constructed from metal nodes and organic linkers via coordination bonds [32]. Their ultra-high surface area, tunable pore structures, and synergistic organic–inorganic functionality render them promising Pt-alternative cathode materials. Indeed, numerous research groups [31,32,56,57,58,59,60,61,62,63,64,65,66,67,68,69,70,71,72,73,74,75,76,77,78,79,80] have extensively investigated MOF-based cathode materials for PFC applications. However, practical implementation is hindered by intrinsic limitations, particularly their low electrical conductivity and limited aqueous stability. To overcome these bottlenecks, research strategies primarily follow two avenues: (1) the direct utilization of MOFs and their conductive composites, and (2) the conversion of MOFs into robust derivatives (e.g., metal/carbon hybrids or porous carbons) that retain structural merits while exhibiting enhanced electrochemical performance.

#### 3.3.1. MOF-Based Cathodes

Direct employment of MOFs exploits their semiconductor-like behavior and pore confinement effects. The band structure of MOFs can be rationally modulated via linker functionalization or defect engineering to extend light absorption and promote charge transfer. Introducing functional groups or lattice defects effectively narrows the bandgap and creates active sites. For instance, Yang’s group [78] reported that NH_2_-functionalized UiO-66 exhibits a narrowed bandgap (~2.8 eV) compared to pristine UiO-66 (~3.7 eV), enabling visible-light response through enhanced ligand-to-metal charge transfer (LMCT). A representative pristine MOF photocathode was developed by Tan et al., who modified a p-type Zn-based MOF with carbon quantum dots (N-Zn-MOF/CQDs) for Cr (VI) reduction. CQDs were first synthesized via an alkali-assisted ultrasonic method using glucose as the carbon source, then incorporated into in situ grown N-doped Zn-MOF on an FTO substrate through solvothermal treatment, and the obtained composite photocathode was paired with a BiVO_4_ photoanode to construct the PFC system for synchronous Cr (VI) removal and power generation. The CQDs acted as electron acceptors and extended the light absorption into the visible region.

To overcome the inherently poor conductivity of pristine MOFs, composites with carbon materials or conductive polymers are frequently constructed. Wang et al. [76] grew ZIF-67 nanosheets directly on carbon felt, creating a porous 3D cathode for simultaneous U (VI) reduction and chlortetracycline hydrochloride oxidation. Benefiting from the enlarged electrochemical active area and facilitated charge transfer, this electrode achieved nearly 100% removal of both pollutants within 60 min and a maximum power density of Benefiting from the enlarged electrochemical active area and facilitated charge transfer, this electrode achieved nearly 100% removal of both pollutants within 60 min and a maximum power density of 580 μWcm^−2^. Similarly, Behera et al. [58] highlighted ZIF-8-based composites and noted that ZIF-8/TiO_2_ and ZIF-8@CQD core–shell structures significantly outperform pristine ZIF-8 in H_2_ evolution and dye degradation due to improved charge separation and upconversion effects. A ZIF-8/rGO composite achieved a photocurrent density over five times higher than that of pure ZIF-8, illustrating the effectiveness of π–π stacking with graphene.

Incorporating conductive polymers into metal–organic frameworks offers another effective strategy for boosting catalytic activity. For instance, Wang et al. [31] constructed a composite of ZIF-67 and polypyrrole (PPy), in which the N–Co coordination selectively directs the ORR along a 2e^−^ pathway, thereby promoting efficient H_2_O_2_ generation. When employed as a cathode, the ZIF-67/PPy composite not only outperformed both Pt and the individual components in the degradation of polyvinyl alcohol but also maintained remarkable stability over four consecutive cycles.

#### 3.3.2. MOF Derivatives as Photocathodes

While compositing with conductive materials effectively enhances the conductivity of MOFs [62], converting them into derivatives through pyrolysis, phosphorization, or sulfidation offers a more fundamental solution to their intrinsic instability. MOF derivatives boast high compositional and structural tunability, which has driven extensive research into their catalytic applications [62]. These derived materials can be constructed into diverse functional systems, including porous carbon-based materials with excellent catalytic activity and metal compounds with metal-like conductivity. The composition and electronic structure of MOF derivatives can be precisely regulated by optimizing precursor design and pyrolysis conditions. Bimetallic MOF precursors have prominent performance advantages: their derived materials can further boost catalytic performance through bimetallic synergy and enhanced interfacial charge separation [69]. Mixed-linker MOFs provide an additional degree of freedom for tailoring the properties of both the parent framework and its corresponding derivatives. The introduction of functional secondary linkers can extend the light absorption range and construct multi-channel charge transfer pathways, thus accelerating electron migration and improving overall catalytic efficiency. Specifically, Priyadarshini et al. employed MIL-53(Fe) as a precursor to fabricate Fe_3_O_4_/MWCNT composite cathodes via pyrolytic derivation, which demonstrated excellent degradation activity toward acridine orange in bio-electro-Fenton systems [66]. On the front of cathode architecture, Qi’s group [67] constructed an S-TiO_2_@MOF-808 heterostructure by coupling sulfur-doped TiO with MOF-808, significantly boosting interfacial charge transfer efficiency in microbial fuel cells. Additionally, a systematic review by Rostami et al. [69] on nanoengineered MOF-derived materials has further elucidated that their tailorable pore structures and densely exposed active sites endow them with considerable promise for the electrocatalytic removal of organic pollutants.

Taken together, MOF-based cathodes exploit the structural programmability and high porosity of pristine MOFs, whereas MOF derivatives overcome the inherent limitations of the parent frameworks by converting into more conductive and stable phases [62]. Linker functionalization, defect engineering, and composite construction with carbons or polymers are key strategies for optimizing both pristine and derived materials [63]. Looking forward, future research should aim at improving long-term stability under realistic operating conditions and developing scalable, green synthesis routes.

### 3.4. Other Cathode Materials

Beyond the extensively studied transition metal compounds, carbon-based materials, and MOFs, the exploration of alternative cathode systems has expanded to include noble metals [26,28,81,82,83,84,85,86,87,88], multicomponent heterojunctions [25,44,89,90], and perovskite-based materials [91,92,93].

**Figure 5:**
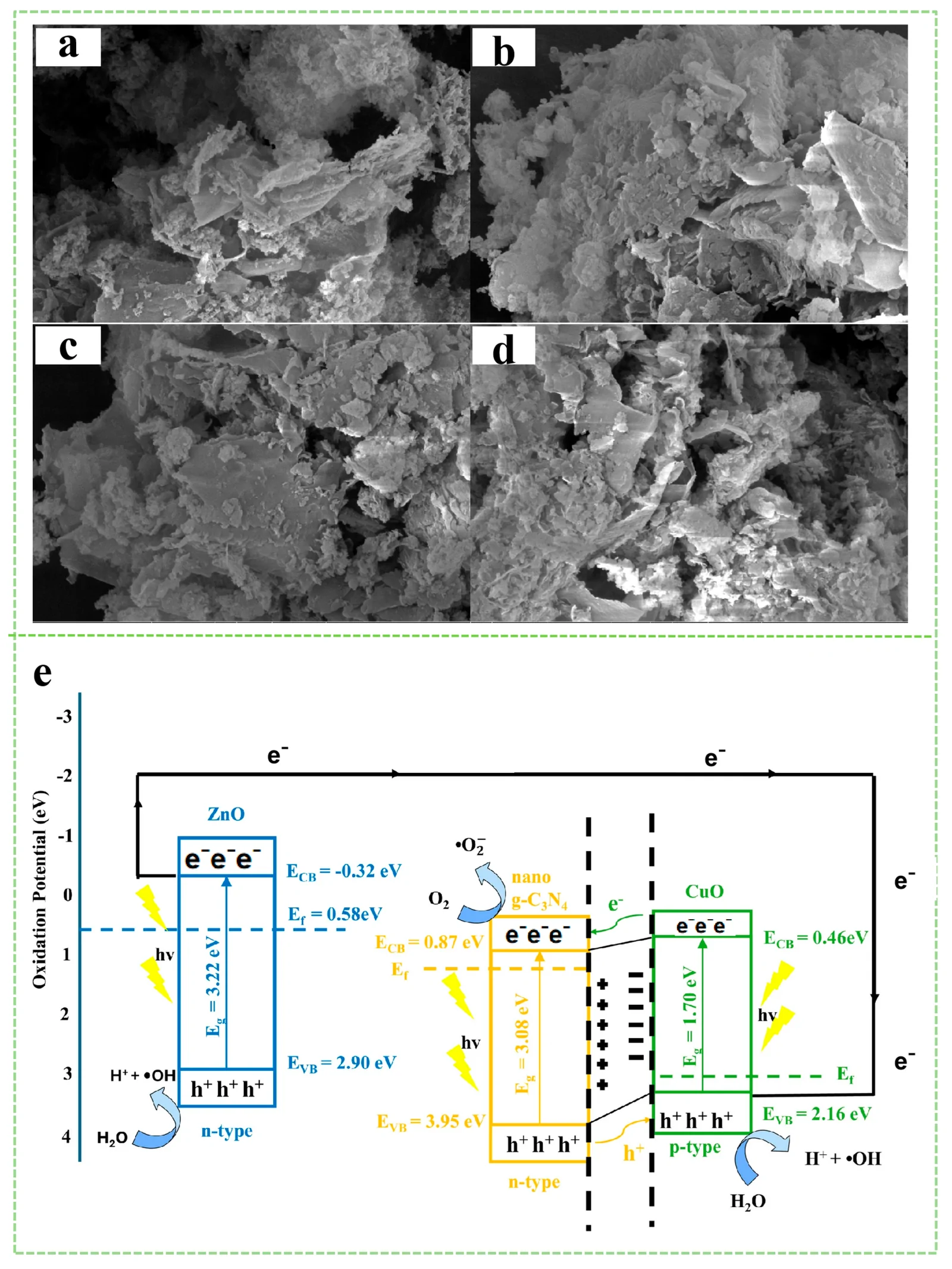
FE-SEM images of (**a**) nano g-C_3_N_4_, (**b**) Cu_0.5_GCN_9.5_, (**c**) Cu_1_GCN_9_ and (**d**) Cu_2_GCN_8_; (**e**) Schematic diagram for mechanism of dual photoelectrodes configurations in PFC system and the possible formation of a heterojunction between the CuO/nano g-C_3_N_4_ composite [90].

Multicomponent heterojunctions combining p-type metal oxides with n-type graphitic carbon nitrides have emerged as a promising class of noble-metal-free photocathodes. A representative example is the CuO/nano g-C_3_N_4_ composite system [90], in which CuO nanoparticles are anchored on exfoliated g-C_3_N_4_ nanosheets to form a rough, flake-like morphology with abundant exposed active sites (Figure 5a–d). The intimate contact between p-type CuO and n-type nano g-C_3_N_4_ creates a p–n junction that establishes an internal electric field, driving photogenerated electrons from the CuO conduction band toward that of nano g-C_3_N_4_ while holes migrate in the opposite direction, thereby markedly suppressing charge recombination (Figure 5e). Under a dual-photoelectrode configuration, the optimized Cu_0.5_GCN_9.5_ photocathode harnesses these separated electrons for efficient oxygen reduction, achieving 98.46% Reactive Red 120 decolorization and a maximum power density of 0.135 μW cm^−2^—167% higher than that of pristine nano g-C_3_N_4_. Such metal-oxide/carbon-nitride heterojunctions illustrate how rational band alignment and interfacial electric fields can simultaneously boost pollutant degradation and electricity generation in PFCs.

Noble metal cathode materials for PFCs cover a variety of specific systems, including platinum-based alloy nanoparticles (e.g., Pt-Co, Pt-Ni alloys), size-tunable silver nanocrystals, gold nanowire array electrodes, palladium nanosheet catalysts, and bimetallic noble metal core–shell structures. As a cost-effective representative option among these systems, silver-based nanostructured materials exhibit satisfactory ORR performance in alkaline electrolytes. The synergistic effect between silver active sites and functional supports can optimize oxygen adsorption behavior and interfacial electron transfer, enabling onset potentials and limiting current densities comparable to commercial Pt/C catalysts. Kuntyi et al. [28] demonstrated that morphology regulation greatly enhances the oxygen reduction catalytic performance of silver-based cathodes and the Ag-Mn synergistic effect enables Ag–MnO_2_/CNT composites to follow a four-electron reaction pathway, while Murugadoss and co-workers [94] prepared silver nanoparticle electrodes with high specific capacitance and excellent cycling stability via a facile chemical reduction method.

To facilitate practical material selection, a holistic comparison of the aforementioned cathode categories is essential. Detailed quantitative metrics are summarized in Supplementary Table S1. Transition metal oxides and sulfides generally offer a favorable balance between catalytic activity and low cost, yet their moderate stability under prolonged illumination remains an ongoing concern [1,21]. Carbon-based materials, particularly carbon felt and graphene composites, excel in electrical conductivity and long-term stability with very low cost, but typically favor the 2e^−^ ORR pathway unless heteroatom-doped or surface-modified [37,50]. Pristine MOFs provide exceptional structural tunability via ligand functionalization and pore engineering; however, their poor aqueous stability and medium cost currently limit immediate practical deployment [32,95]. MOF-derived materials and single-atom catalysts can overcome these stability limitations while maintaining high selectivity, albeit at the expense of increased synthesis complexity [43]. Noble metals remain the benchmark for activity and stability, yet their very high cost and scarcity restrict large-scale wastewater treatment applications [34,96]. Conducting polymers introduce unique mechanical flexibility and dopant-tunable conductivity, though their moderate stability in complex wastewater matrices requires further evaluation [29,97]. Ultimately, cathode selection represents a multi-criteria optimization problem involving performance, stability, and cost. 

## 4. Applications of PFCs with Engineered Cathodes

The cathode is a critical component determining the overall performance and application scope of PFCs. Depending on the cathode material and reaction pathway, PFCs can be designed for wastewater treatment, heavy metal detoxification, and simultaneous electricity generation. Before delving into specific scenarios, it is essential to rationalize the cathode design strategy from the perspective of ORR pathway selectivity. The cathodic ORR can proceed via two distinct routes with profoundly different application implications. The four-electron pathway extracts the full chemical potential of oxygen, delivering higher open-circuit voltage and power density; it is therefore the preferred route when electricity generation is the primary objective [96]. In contrast, the two-electron pathway generates hydrogen peroxide, a valuable in situ oxidant that can be activated to produce •OH and SO_4_•^−^ for advanced oxidation of recalcitrant organics [29,33,50]. The selectivity between these two pathways is not intrinsic but can be deliberately tuned by material engineering. Cathode design can switch ORR selectivity between energy recovery (4e^−^, e.g., PPy, *n* = 3.39 [95]) and in situ oxidation (2e^−^, e.g., ZIF-67/PPy, *n* = 2.32 [95]); carbon felt [50] and PPy/MWCNTs/Fe_3_O_4_ [29]), with the latter maximizing H_2_O_2_ generation up to 11.5 mg L^−1^. Conversely, high-performance energy-recovery systems such as Pt-black [96] and SnS_2_/CF [39] cathodes favor the 4e^−^ pathway to maximize power output. Consequently, a rational design principle emerges: cathodes for wastewater treatment requiring pollutant mineralization should be tailored for 2e^−^ ORR selectivity or coupled oxidant activation, whereas cathodes targeting electricity generation should pursue 4e^−^ ORR selectivity through strong O_2_ adsorption and rapid O–O bond cleavage. The following subsections are organized according to these distinct application objectives.

### 4.1. Organic Pollutants Removal

Given that the complete mineralization of organic pollutants typically relies on the in situ generation and subsequent activation of H_2_O_2_, cathodes in this category are generally engineered to favor the 2e^−^ ORR pathway or cathodic oxidant activation [5,29,33]. The degradation of organic pollutants is the most extensively studied PFC application [13,20,98,99]. Cathode materials govern the generation of reactive oxygen species (ROS) through ORR and oxidant activation, directly influencing pollutant removal efficiency.

#### 4.1.1. Dye Degradation

**Figure 6:**
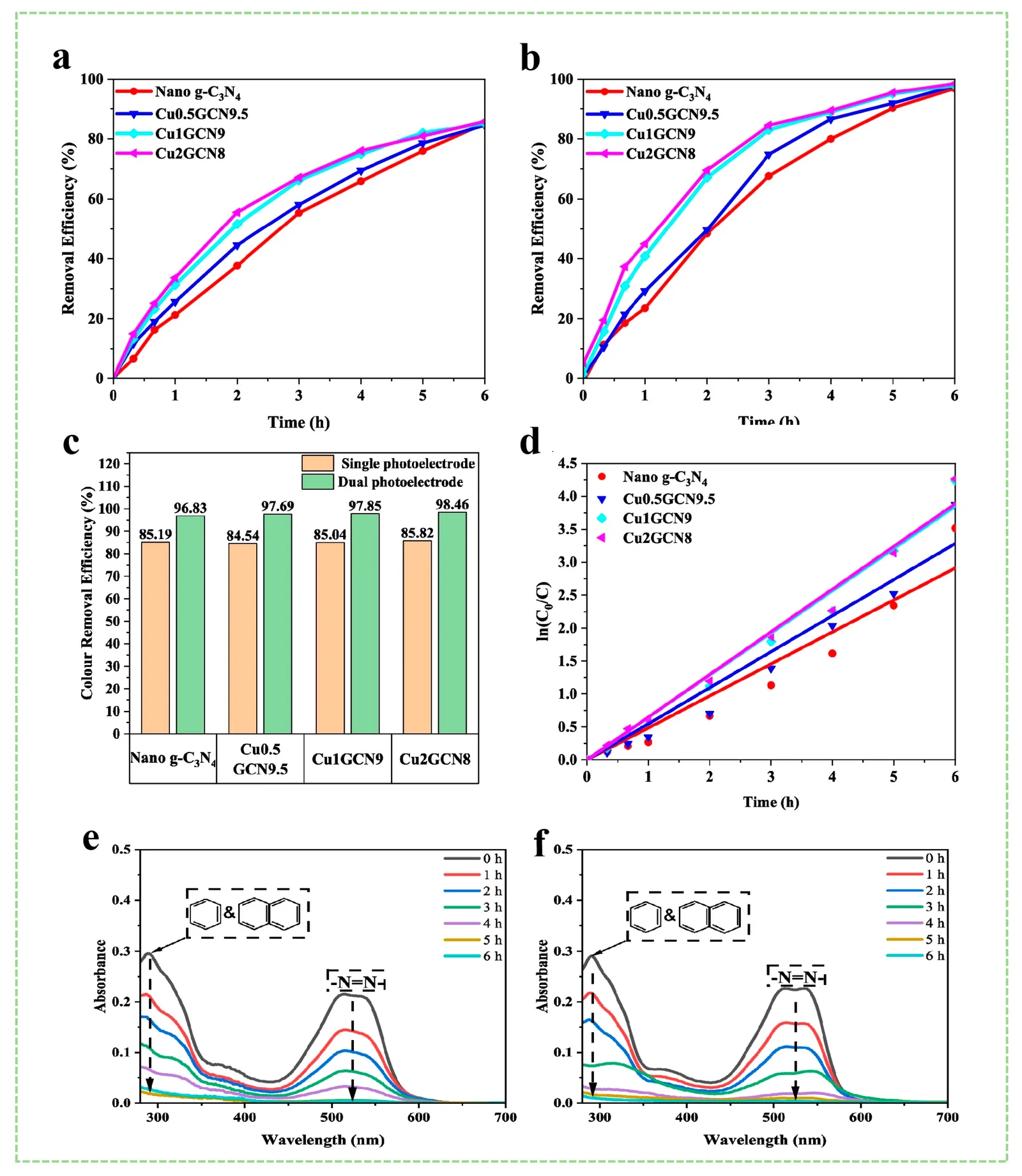
Decolorization of RR120 (10 mg L^−1^) with different cathodes in PFC systems: (**a**) single-photoelectrode and (**b**) dual-photoelectrode time profiles; (**c**) final efficiency comparison. (**d**) Kinetic degradation fits for dual-electrode configurations. (**e**,**f**) Time-dependent UV–Vis spectra for Cu_0.5_GCN_9.5_ cathode under single- (**e**) and dual-electrode (**f**) modes, showing absorbance decay in line with removal trends [90].

While noble-metal cathodes such as Pt-black achieve exceptional power densities through the efficient 4e^−^ ORR [96], many carbon-based composite cathodes in dye-degradation systems operate via the 2e^−^ route to generate H_2_O_2_ for electro-Fenton oxidation [50,54]. Dyes are common model pollutants for evaluating PFC performance. Several recent studies have explored diverse cathode materials, yielding a wide range of degradation and power performance. For instance, a PANI/MWCNTs cathode delivered 98.38% AB113 removal but only 750 μWcm^−2^ [97]. In another approach, Sb:SnO_2_ thin films with a narrowed band gap (from 3.87 to 3.40 eV) were fabricated by Maharana et al. [100], achieving 55% methylene blue degradation in 90 min and a much higher power density of 2250 μW cm^−2^. Even greater power (4240 μW cm^−2^) was attained during methyl orange decomposition using a Pt black cathode [96]. To balance efficiency and energy recovery, a MnO_2_/GO composite coupled with a BiVO_4_/WO_3_ photoanode was developed by Ouyang’s group [37]; this configuration degraded ~95% Rhodamine B in 6 h and increased the power density by 2.2 times relative to pristine MnO_2_. In a representative study, Khor et al. [90] systematically evaluated CuO/nano g-C_3_N_4_ composites as photocathodes for Reactive Red 120 degradation under single and dual photoelectrode configurations. As shown in Figure 6a,b, the dual-photoelectrode setup markedly accelerated decolorization kinetics compared to the single-photoelectrode counterpart across all compositions, with Figure 6c revealing that Cu_2_GCN_8_ achieved the highest removal efficiency of 98.46% after 6 h. Pseudo-first-order kinetic analysis (Figure 6d) further confirmed this enhancement, showing that the apparent rate constant increased with CuO loading and reached 0.6477 h^−1^ for Cu_2_GCN_8_ under dual illumination. These performance gains were corroborated by time-resolved UV-Vis spectra (Figure 6e,f), where the progressive attenuation of the azo bond (~536 nm) and aromatic ring (~300 nm) absorption peaks verified the effective molecular decomposition of the dye.

Beyond conventional photoactivation, a double Z-scheme g-C_3_N_4_/ZnO/Bi_4_O_5_Br_2_ cathode was found to degrade 93% of RhB even under dark conditions, driven by O_2_•^−^ generation, as reported by Yu et al. [44]. For selective oxidation of SIPX, a BPO/BP-BMO composite was designed by Yin and co-workers [9], achieving 92% degradation within 90 min via ^1^O_2_ attack on the –O–CSS– bond. In the field of covalent organic frameworks, the TP-oBPy COF developed by Gao et al. [101] exhibited >98% sulfadiazine degradation in 50 min and an outstanding H_2_O_2_ production rate of 6776 μmol·g^−1^·h^−1^, with ^1^O_2_ and O_2_•^−^ as the dominant reactive species.

#### 4.1.2. Antibiotic Degradation

Antibiotics represent a class of persistent aquatic pollutants that pose significant ecological risks, necessitating highly efficient mineralization strategies. In PFC systems, cathodes capable of driving the 2e^−^ ORR or activating PMS/periodate are particularly effective. These pathways generate potent oxidants—such as H_2_O_2_, SO_4_•^−^, or singlet oxygen (^1^O_2_)—that drive the breakdown of recalcitrant antibiotic molecules into harmless end-products [5,25].

Recent advances have demonstrated the efficacy of various heterojunction photocathodes in this domain. For instance, a piezoelectric BiOBr/g-C_3_N_4_ cathode was designed by Yu and co-workers [25], achieving 93.41% tetracycline (TC) degradation and 85.61% TOC removal, with electron paramagnetic resonance confirming that •OH and O_2_•^−^ were the dominant reactive species. Employing a coupled photoanode–cathode configuration, Hajiali et al. [42] paired a CuO/Cu_2_O/Cu mesh cathode with a ZnO/Bi_2_MoO_6_/MIL-101(Fe) photoanode, which raised the TC degradation efficiency to 95.1%. A significant enhancement in degradation kinetics was reported by Wu et al. [5], who introduced a Bi_4_O_5_I_2_/TiO_2_/PMS system that exhibited a 17-fold higher rate constant (0.0853 min^−1^), a power density of 990 μW cm^−2^, three identified mineralization pathways, and intermediates with lower toxicity than TC itself.

Beyond conventional radical-mediated oxidation, emerging studies highlight the role of non-radical and electron-transfer mechanisms. A Ti_3_C_2_/g-C_3_N_5_ heterojunction was constructed by Haroon and colleagues [102], in which the 3% MXCN composite achieved 97% TC degradation within 90 min (k = 0.0314 min^−1^); the proposed degradation pathways and toxicity assessment confirmed that the intermediates were less toxic than the parent compound. For ciprofloxacin treatment, a Bi-MOF/g-C_3_N_4_ Z-scheme heterojunction was fabricated by Vanaja et al. [103], delivering 99.8% CIP degradation (k = 6.52 × 10^−3^ min^−1^).

**Figure 7:**
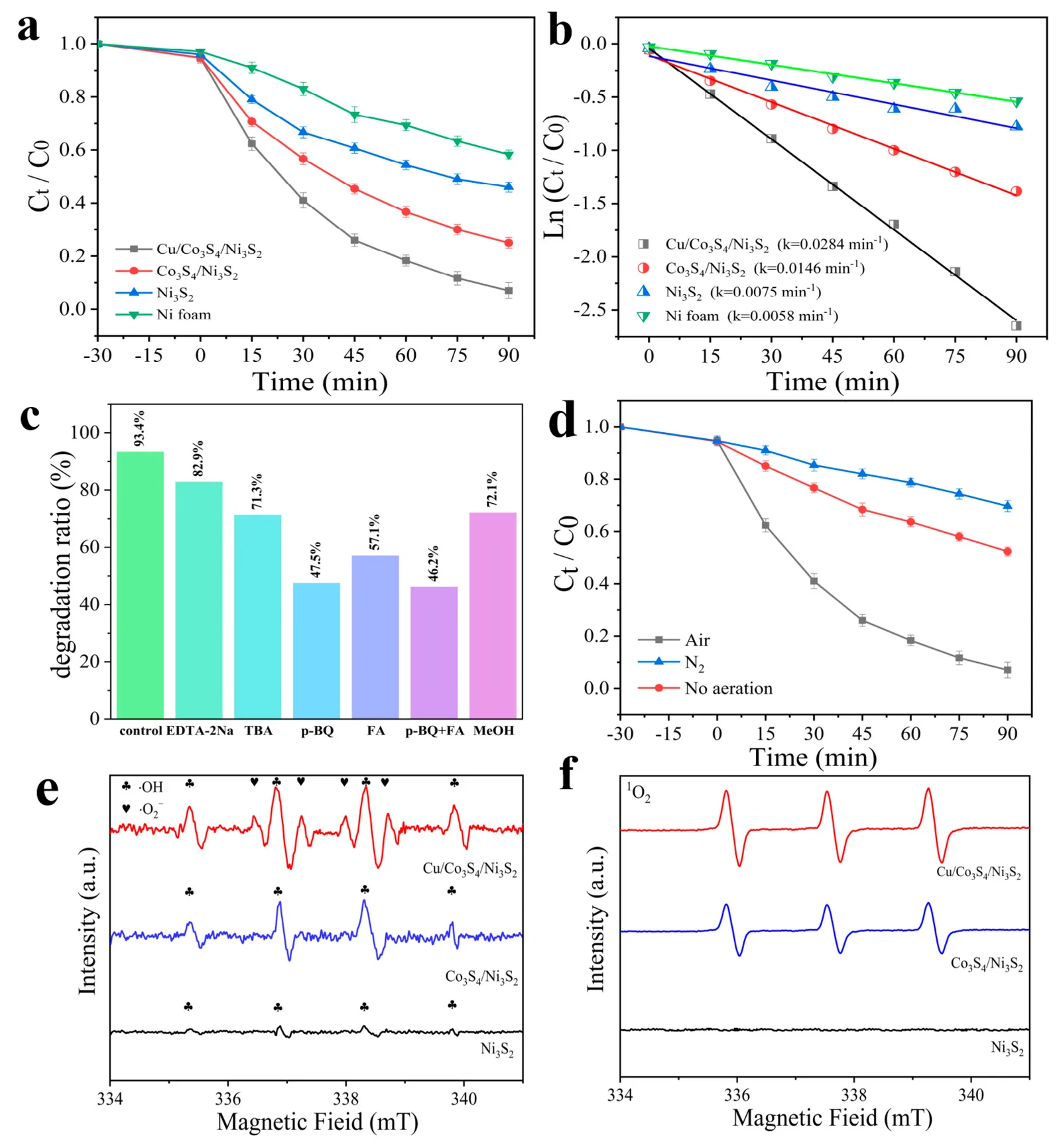
(**a**) Degradation of rifampicin (20 mg L^−1^) and (**b**) corresponding pseudo-first-order kinetics in PFCs with different cathodes. (**c**) Effects of radical scavengers on RFP removal in the Cu/Co_3_S_4_/Ni_3_S_2_ system. (**d**) RFP degradation under air, N_2_, and no-aeration conditions. EPR spectra of (**e**) DMPO–•OH/•O_2_^−^ and (**f**) TEMP–^1^O_2_ adducts over Ni_3_S_2_, Co_3_S_4_/Ni_3_S_2_, and Cu/Co_3_S_4_/Ni_3_S_2_ cathodes [45].

A detailed mechanistic investigation into multi-radical generation was conducted by Wang et al. [45] using a Cu/Co_3_S_4_/Ni_3_S_2_ cathode for rifampicin (RFP) degradation. As shown in Figure 7a, the system achieved 93.4% RFP removal within 90 min (TOC removal 70.1%), outperforming Co_3_S_4_/Ni_3_S_2_ (75.2%), Ni_3_S_2_ (54.3%), and bare Ni foam (42.2%). Kinetic analysis (Figure 7b) yielded a rate constant of 0.0284 min^−1^, nearly twice that of the undoped counterpart. Quenching experiments (Figure 7c) revealed that •O_2_^−^, ^1^O_2_, and •OH markedly suppressed degradation, whereas methanol showed an effect comparable to tert-butanol, ruling out SO_4_•^−^. Comparative tests under different aeration conditions (Figure 7d) further confirmed the pivotal role of oxygen: degradation dropped to 48.7% without aeration and sharply to 30.4% under N_2_, indicating that cathodic O_2_ reduction is the primary source of reactive species. EPR spectra (Figure 7e,f) subsequently verified strong •OH, •O_2_^−^, and ^1^O_2_ signals in the Cu-doped system, demonstrating that Cu incorporation enhances multi-radical generation via accelerated ORR kinetics for efficient antibiotic mineralization.

For recalcitrant organics such as quinoline and polyvinyl alcohol, the synergy between 2e^−^ ORR-generated H_2_O_2_ and external oxidant activation (e.g., persulfate) proves critical in achieving high removal efficiencies [29,95]. Various modified cathode materials have recently been employed in photocatalytic fuel cell systems for pollutant removal and resource recovery. In terms of quinoline degradation, the work by Parvizi and co-workers [29] utilized a PPy/MWCNTs/Fe_3_O_4_ cathode coupled with CuFe-LDH-activated persulfate, attaining 98.2% quinoline removal and 71.6% COD elimination. For polyvinyl alcohol (PVA) degradation, a ZIF-67/PPy cathode developed by Wang’s group [95] outperformed both Pt and the individual components; in a separate application, the same team also demonstrated simultaneous oxidation of chlortetracycline hydrochloride (CTC-HCl) and reduction in U (VI) using a ZIF-67/carbon felt cathode, achieving nearly 100% removal of both pollutants within 60 min.

### 4.2. Heavy Metal and Radionuclide Reduction

In contrast to the radical-mediated oxidation mechanisms prevalent in organic pollutant degradation, the remediation of heavy metals and radionuclides in PFCs relies predominantly on direct cathodic electroreduction or surface-mediated redox cycling rather than selective ORR pathways [26,32]. Capitalizing on the strong reducing power of photogenerated electrons, PFC cathodes can convert highly toxic, mobile, high-valence species into less hazardous or recoverable low-valence forms. This approach offers an energy-efficient alternative to conventional chemical precipitation or standalone electrochemical reduction. This section delineates recent progress in heavy metal and radionuclide reduction.

#### 4.2.1. Heavy Metal Reduction

Hexavalent chromium (Cr (VI)) is one of the most common toxic heavy metal pollutants, and its reduction to trivalent chromium (Cr (III)) greatly reduces both toxicity and environmental mobility. PFC cathodes have been designed to achieve efficient Cr (VI) reduction through direct electron transfer or surface-mediated redox cycling. To address the high cost and sluggish kinetics of Pt cathodes, Sun’s group [26] developed an Ag@Fe_2_O_3_ core–shell cathode, where the Fe_2_O_3_ shell provides redox sites and the Ag core ensures rapid charge transport. This cathode achieved a Cr (VI) reduction rate constant of 0.058 min^−1^—twice that of Pt foil—offering a simple non-Pt paradigm. Building on this, Tan et al. [32] introduced photoresponse via a CQD-modified N-Zn-MOF photocathode. When coupled with a BiVO_4_ photoanode, the PFC system achieved efficient Cr (VI) reduction. Under optimized conditions (pH 2), 100% Cr (VI) removal was obtained within 120 min [30], and the system exhibited good stability over five consecutive cycles.

Beyond detoxification, PFC systems offer a unique advantage in the resource recovery of heavy metals, exemplified by Cu^2+^ reduction. Lam et al. [34] developed a MoO_3_/ZnO/Zn photoanode with a Pt/C cathode, achieving 93.5% Cu^2+^ removal and 89.5% COD removal within 240 min at optimized conditions (200 mg L^−1^ Cu^2+^, pH 3), with a maximum power density of 7 μW cm^−2^. Liu et al. [27] employed a TiO_2_@CoOx photoanode with a graphite cathode; under N_2_ purging to suppress oxygen competition, the system attained 91% Cu^2+^ removal within 12 h, with XRD confirming metallic Cu as the exclusive reduction product. In contrast, dissolved oxygen competed for electrons, lowering Cu^2+^ removal to 65% while enhancing power output and anodic oxidation. The practical feasibility was validated with real copper plating wastewater, achieving 85.0% Cu^2+^ and 73.6% COD removal [34]. These findings establish PFC cathodes as an effective platform for simultaneous Cu^2+^ recovery, organic degradation, and energy generation.

#### 4.2.2. Radioactive Element Reduction

Uranium, typically present as highly soluble hexavalent U (VI) species in contaminated water, can be reduced to insoluble tetravalent U(IV) and subsequently deposited on the cathode surface, enabling simultaneous detoxification and recovery. PFC cathodes for U (VI) reduction require both strong reducing capability and abundant surface-active sites for U (IV) immobilization.

One study by Cao et al. [3] constructed a Co-N_x_/C@MXene cathode. Characterization confirmed a synergistic reduction pathway. The cathode achieved 98.1% U (VI) and 97.1% TCH removal simultaneously, with >93% activity retained after 10 cycles, demonstrating excellent stability. Subsequently, Wang et al. [39] employed a SnS_2_/CF cathode in a hybrid tandem PFC. The wool-ball-like SnS_2_ nanostructure facilitated mass transport and provided abundant active sites. The system achieved 89.95% surface uranium as U (IV) and a power density of 6490 μWcm^−2^ under real sunlight. In a later study, the same team [76] developed a ZIF-67/CF cathode. The porous 3D nanosheet network enlarged the active area and reduced charge-transfer resistance. Within 60 min, nearly 100% U (VI) was reduced to U (IV). After 8 cycles, removal efficiencies remained above 90%. These advances underscore the immense potential of rationally designed PFC cathodes for the simultaneous detoxification and resource recovery of radionuclides from contaminated water.

### 4.3. Wastewater Treatment Coupled with Electricity Generation

This subsection focuses on energy-recovery-oriented systems, where the 4e^−^ ORR pathway is generally favored to maximize power density and electron utilization efficiency [39,96].

#### 4.3.1. High Power Density Systems

High-performance PFC systems aim to harness the full thermodynamic potential of oxygen reduction, typically relying on efficient 4e^−^ ORR pathways to minimize overpotential. Early breakthroughs were achieved using noble-metal catalysts: Liao et al. [96] achieved a maximum power density of 4240 μW cm^−2^ using a Pt-black cathode, representing one of the highest values reported for visible-light-driven PFC. A study by Wang [39] reported a power density of 6490 μW cm^−2^ under real sunlight using a SnS_2_/CF cathode in a hybrid tandem PFC, demonstrating the potential of metal sulfide/carbon composites for practical solar-driven applications. Wang and co-workers [76] achieved a power density of 580 μWcm^−2^ with a ZIF-67/CF cathode, which was 5.58 times that of state-of-the-art PFCs at the time. Pushing the boundaries further, ZnO/CF cathodes were developed for hybrid tandem PFCs, enabling concurrent uranium removal and organic degradation with a power density of 1310 μW cm^−2^, highlighting the versatility of metal oxide/carbon composites for complex wastewater treatment [104].

#### 4.3.2. Moderate Power Density with Coupled Degradation

**Figure 8:**
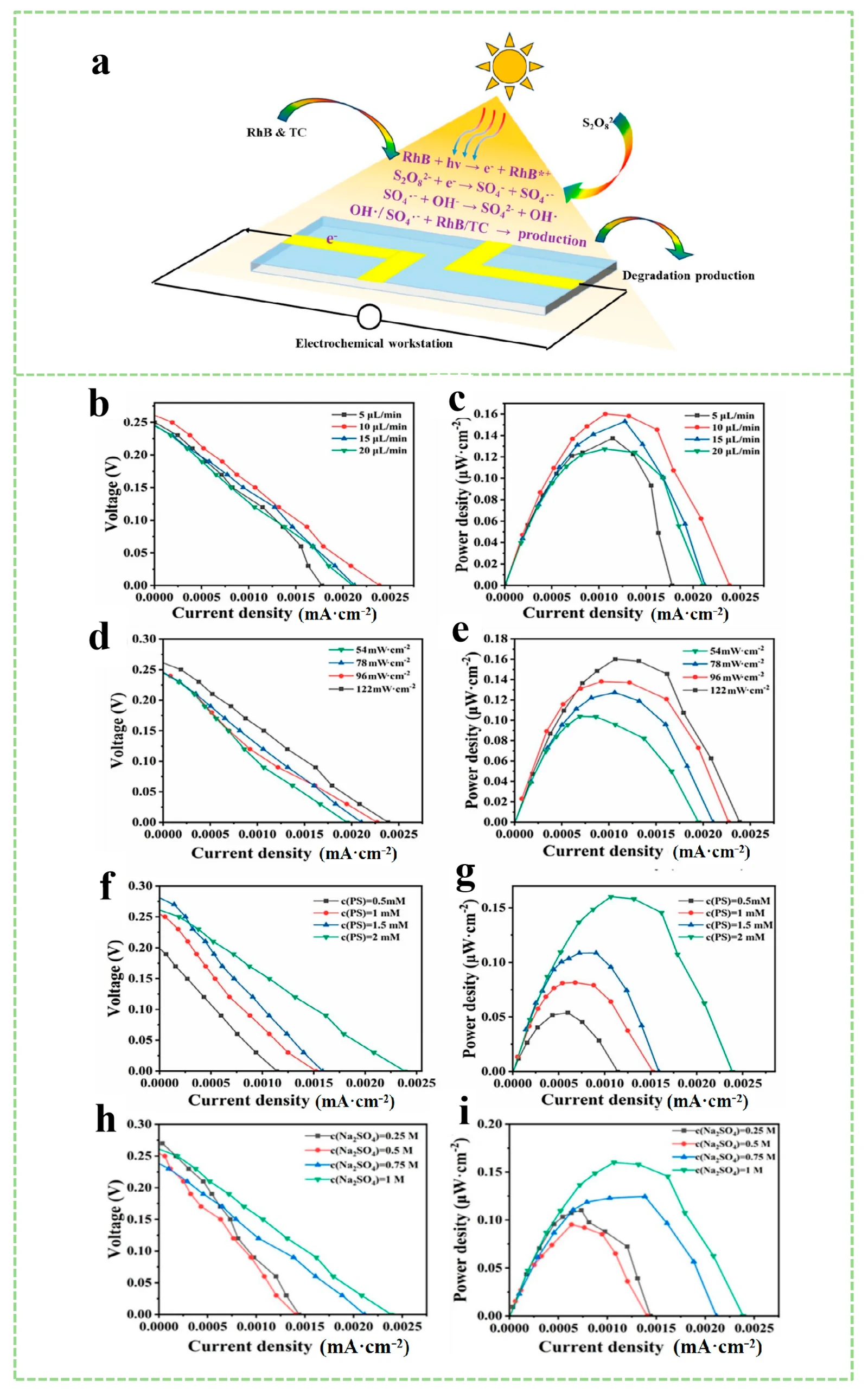
(**a**) The reaction mechanisms of PFC systems. Polarization curves (**left column**) and corresponding power density curves (**right column**) of the microfluidic PFC under varying operational parameters: (**b**,**c**) flow rate, (**d**,**e**) light intensity, (**f**,**g**) persulfate concentration, and (**h**,**i**) sodium sulfate concentration [106].

Systems in this category often strike a balance between energy recovery and pollutant removal, where cathodes may simultaneously sustain moderate power output via the 4e^−^ ORR while contributing 2e^−^ ORR-derived H_2_O_2_ or activated radicals for coupled degradation [37,105]. Zhou et al. [106] further demonstrated that reactor engineering and operational parameters critically govern the power–degradation balance in microfluidic PFCs with gold-plated cathodes. Figure 8a schematically depicts the charge-transfer and radical-generation cascade in the microfluidic PFC: photoexcited RhB injects electrons into the gold-plated cathode, where they reductively activate persulfate to produce SO_4_^−^· and ·OH for tetracycline mineralization. Systematic optimization revealed that a flow rate of 10 μL/min (Figure 8b,c), a light intensity of 122 mW·cm^−2^ (Figure 8d,e), a persulfate concentration of 2 mM (Figure 8f,g), and a Na_2_SO_4_ concentration of 1 M (Figure 8h,i) collectively afforded the highest power density of ~0.16 μW·cm^−2^ and a short-circuit current density of 0.00239 mA·cm^−2^. Under these conditions, the cathodic reduction of persulfate by photogenerated electrons was maximized, tripling the power output relative to suboptimal PS loading while concurrently boosting tetracycline degradation from 35.16% to 60.78%. This case underscores that even in noble-metal-cathode microfluidic systems, fine-tuning of electrolyte composition and fluid dynamics is essential to approach the performance ceiling dictated by cathodic oxidant activation.

Several design principles emerge from the literature for maximizing power output: (1) reducing cathode charge transfer resistance through conductive carbon composites or direct growth on conductive substrates [39,43]; (2) engineering ORR selectivity to favor the 4e^−^ pathway for higher energy efficiency or the 2e^−^ pathway for coupled advanced oxidation [95]; (3) constructing heterojunctions to suppress carrier recombination and preserve strongly reducing electrons for ORR [25]; and (4) coupling with external oxidants (persulfate, periodate) to enhance radical generation while maintaining power output [29,88].

## 5. Challenges and Future Perspectives

Although the design and application of PFC cathode materials have made the above breakthrough progress, there are still multi-dimensional challenges from laboratory research to large-scale practical application. Future systematic innovation needs to be carried out around three levels: material, mechanism and device.

### 5.1. Challenges

(1) Material stability and cost balance: Non-noble-metal cathodes suffer from stability bottlenecks including photocorrosion of oxides/sulfides [10,11], structural collapse of MOFs [34,95], and active-component detachment. Current cycle stabilities are typically <20 cycles [32,39,95], far below the >1000 h benchmark for industrial systems. Laboratory fabrication methods [30] incur high areal costs, precluding economic viability.
  More specifically, the stability bottlenecks vary distinctly across material classes. For transition-metal oxides and sulfides, narrow-bandgap semiconductors such as Cu_2_O suffer from severe photocorrosion driven by photogenerated holes oxidizing Cu^+^ to Cu^2+^, while MnO_2_ and Fe-based oxides are limited by intrinsically low conductivity (10^−5^–10^−6^ S cm^−1^) [30,37]. Carbon-based cathodes, though chemically robust in principle, undergo oxidative corrosion in aerated electrolytes during long-term operation, leading to active-site loss and performance decay [50,54]. For MOF-derived materials, structural collapse and active-site leaching are frequently observed in complex wastewater matrices [76,95]. These material-specific failure modes are insufficiently addressed by generic stabilization strategies and require targeted solutions.
(2) Interfacial mechanism is not clear: Most current studies rely on offline characterization methods to analyze reaction mechanisms, lacking in situ dynamic monitoring of interfacial charge transfer, ORR intermediate adsorption states, and pollutant reduction pathways; consequently, the structure–activity relationships between cathode architectures and their electrocatalytic performance remain elusive. Furthermore, the influence of realistic wastewater chemistries—including coexisting heavy metals, humic acids, halide ions, and inorganic salts—on cathode surface active sites and interfacial charge transfer processes is still poorly understood. As highlighted in Section 4.1 and Section 4.2, these components can fundamentally alter reaction pathways: dissolved oxygen competes with high-valence pollutants for photogenerated electrons, creating a trade-off between power output and pollutant removal efficiency [27], whereas abundant Cl^−^ in high-salt matrices rapidly scavenges primary radicals and redirects oxidation mechanisms toward reactive chlorine species, reconstructing degradation pathways without altering the cathode material itself [22,105]. The lack of systematic investigations under authentic wastewater conditions therefore leads to a significant performance gap between laboratory optimization and practical field deployment.
(3) Scale-up barriers from laboratory to industrial deployment. The leap from laboratory-scale electrodes (typically <10 cm^2^) to industrially relevant dimensions (>1 m^2^) introduces multidimensional engineering bottlenecks spanning materials processing, reactor design, and operational logistics. Current proof-of-concept studies predominantly rely on H-type or dual-chamber configurations [17,23,95], which obscure scale-dependent limitations: enlarged electrode areas inevitably suffer from non-uniform photon distribution, aggravated ohmic losses, and uneven current density distribution, all of which diminish faradaic efficiency and power output. Furthermore, laboratory fabrication protocols—such as drop-casting or small-batch hydrothermal synthesis—lack the reproducibility and throughput needed for uniform large-area coating, resulting in batch-to-batch variability and compromised mechanical adhesion of active layers on expanded conductive substrates. At the reactor level, the absence of modular, stackable architectures and continuous-flow designs necessitates costly system shutdowns for electrode replacement, while unoptimized hydrodynamics at >1 m^2^ scales impose severe mass-transfer constraints and pressure-drop penalties. Consequently, scalable manufacturing routes (e.g., roll-to-roll spray pyrolysis, blade coating) [100] and computational fluid dynamics-aided reactor engineering must be co-developed to close the manufacturing and techno-economic gaps between benchtop demonstrations and practical deployment.

### 5.2. Future Development Directions

(1) High-stability low-cost cathode material design: For the stability bottleneck, the stability of the cathode can be improved through interface engineering (such as constructing S-scheme heterojunctions to inhibit photocorrosion [25], surface coating of MOF materials to improve water stability [34]) and defect engineering (introducing appropriate defects to enhance structural stability [39]); for the cost problem, the loading of noble metals can be reduced by developing single-atom catalysts, or the performance of low-cost materials (biomass carbon [53], natural minerals) can be improved through modification, and low-cost large-scale preparation processes such as blade coating, roll-to-roll spray pyrolysis [100], and flame-assisted synthesis can be developed to reduce the areal cost of cathodes. Equally important, standardized accelerated aging protocols under real wastewater matrices must be established, targeting >1000 h continuous operation with <10% power-density decay.
(2) In situ mechanism characterization and relationship construction: Develop in situ characterization technologies such as in situ Raman spectroscopy, in situ XPS, transient absorption spectroscopy and operando electron microscopy, which can dynamically monitor the interfacial charge transfer process, intermediate product evolution and active site change during the reaction, combined with DFT theoretical calculations, to accurately establish the structure-activity relationship between cathode material electronic structure, interface properties and performance, and provide theoretical guidance for material design.
(3) Modular reactor engineering and bio-hybrid integration for industrial translation. Scalable manufacturing requires continuous routes [100] and low-cost carbon substrates [53]. Modular, stackable architectures should replace conventional H-type cells, enabling plug-and-play electrode replacement without costly system shutdowns. Computational fluid dynamics (CFD) modeling should be employed to optimize flow-field designs and alleviate mass-transfer limitations as electrode dimensions scale up. Standardized long-term stability protocols under realistic wastewater matrices are urgently required to benchmark cathode durability [30,42]. Additionally, bio-hybrid integration strategies offer a promising route to mitigate membrane fouling while enhancing the degradation of recalcitrant pollutants. Ultimately, comprehensive techno-economic analyses at pilot scale are imperative to bridge the gap between laboratory demonstrations and industrial implementation.
(4) Data-driven intelligent design: Given the vast compositional and structural design space spanning transition-metal oxides, carbon allotropes, MOFs, and their hybrids, high-throughput computational screening—combining DFT calculations with machine learning—should be employed to predict optimal band alignments, ORR intermediate adsorption energies, and pollutant reduction pathways. Such in silico guidance can drastically accelerate the discovery of application-specific cathode materials and reduce reliance on empirical trial-and-error [30,31].

In general, the development of high-performance PFC cathode materials provides a new technical path for the synergistic realization of wastewater resource treatment and new energy supply. Through multi-level innovation from material design, mechanism analysis to device optimization, PFC technology is expected to achieve large-scale practical application in the future, and provide support for the construction of a new system of environmental governance and new energy.

## 6. Conclusions

This review systematically summarizes cathode materials for PFC, focusing on their design strategies, reduction mechanisms, and wastewater treatment applications. The cathode governs electron transfer and interfacial reactions, including oxygen reduction, direct pollutant electroreduction, and oxidant activation. Cathodic materials are classified into transition metal oxides/sulfides, carbon-based materials, metal–organic frameworks and their derivatives, each with distinct trade-offs among activity, stability, and cost. Tailored cathodes enable effective degradation of organic pollutants, reduction in heavy metals, and immobilization of radionuclides, while simultaneously generating electricity. Future research should prioritize long-term stability, scalable synthesis, and multi-functional cathode integration to bridge the gap between fundamental studies and practical applications.

## Data Availability

No new data were created or analyzed in this study.

## Supplementary Materials

The following supporting information can be downloaded at: https://www.mdpi.com/article/10.3390/nano16160995/s1, Table S1: Comparative summary of representative PFCs utilizing various cathode materials.
