# Supplementary material for: Cathode Materials for Photocatalytic Fuel Cells: Design Strategies, Reaction Mechanisms, and Wastewater Treatment Applications
Source: Nanomaterials (Basel). 2026 Aug 12;16(16):995. doi: 10.3390/nano16160995 (PMC13515834; doi:10.3390/nano16160995)
Supplement: Supplementary file 1 [file nanomaterials-16-00995-s001.zip › nanomaterials-4477240-supplementary.pdf]

Table S1 Comparative summary of representative PFCs utilizing various cathode materials.

Category	Cathode material	Target pollutant / Electrolyte	Unified power density (μW cm⁻²)	Cost	Selectivity	Ref.
**Transition metal oxides**	MnO_2GO	—	66.2	Low	Tunable (2e⁻/4e⁻ ORR)	[1]
	CuO/Cu_2OCu mesh	Tetracycline	30.58	Low	Tunable (2e⁻/4e⁻ ORR)	[2]
	$BiOAc_{1-x}Br_x/BiOBr$	Tetracycline	714	Low	Tunable (2e⁻/4e⁻ ORR)	[3]
	Sb: SnO_2 thin film	Methylene blue	2250	Low	Tunable (2e⁻/4e⁻ ORR)	[4]
	$Bi_4O_5I_2/TiO_2/PMS$	Tetracycline / PMS	990	Low	Tunable (2e⁻/4e⁻ ORR)	[5]
	Si NWs/$ZnFe_2O_4$/Ag	—	6.81	Low	Tunable (2e⁻/4e⁻ ORR)	[6]
	SnS_2/CF	TCH + U(VI) / 0.1 M Na_2SO_4	6490	Low	Strong reduction potential	[7]
**Single-component carbon**	Carbon felt (CF)	Amaranth	3.56	Very low	Typically favors 2e⁻ ORR	[8]
**Carbon composites**	g-$C_3N_4/Fe_2O_3/MoS_2$/Ti	Rhodamine B	13.26	Low–Medium	Switchable ORR pathway via component selection	[9]
**Pristine MOFs**	ZIF-67/CF	U(VI) + CTC·HCl	580	Medium	Ligand-tunable bandgap and pore confinement	[10]
	Bi-MOF/g-C_3N_4	Ciprofloxacin	1800	Medium	Ligand-tunable bandgap and pore confinement	[11]
	N-Zn-MOF/CQDs	Cr (VI)	1350	Medium	Ligand-tunable bandgap and pore confinement	[12]
**Noble metals**	Pt-black	Methyl orange	4240	Very high	Dominant 4e⁻ ORR (Pt); tunable via alloying/support	[13]
	Pt/C	Cu^{2+} + phenol	7	Very high	Dominant 4e⁻ ORR (Pt); tunable via alloying/support	[14]
**Conducting polymers**	PANI/MWCNTs	AB113	750	Medium	Dopant-tunable conductivity and flexibility	[15]
	PPy/MWCNTs/Fe_3O_4	Quinoline / persulfate	760	Medium	Dopant-tunable conductivity and flexibility	[16]

Reference

1. Ouyang, K., et al., *A novel visible-light responsive photocatalytic fuel cell with a highly efficient $BiVO_4/WO_3$ inverse opal photoanode and a MnO_2/graphene oxide nanocomposite modified cathode.* International Journal of Hydrogen Energy, 2019. **44**(14): p. 7288-7299.

2. Hajiali, M., M. Farhadian, and S. Tangestaninejad, *Enhance performance ZnO/ Bi_2MoO_6/ MIL-101(Fe) grown on fluorine-doped tin oxide as photoanode and CuO/ Cu_2O based on Cu mesh photocathode in the photocatalytic fuel cell.* Energy Conversion and Management, 2022. **269**: p. 116137.

3. Yu, T., et al., *Fabrication of a novel Z-S-scheme photocatalytic fuel cell with the Z-scheme TiO_2/GO/g-*

C_3N_4 photoanode and S-scheme BiOAc1−Br /BiOBr photocathode for TC degradation. Journal of Materials Science & Technology, 2024. **188**: p. 11-26.

4. Maharana, G., et al., *Sustainable dual usage of spray-pyrolyzed Sb:SnO_2 thin films for pollutant degradation and power generation.* Journal of Materials Chemistry A, 2026. **14**(19): p. 11614-11640.
5. Wu, H., et al., *Peroxymonosulfate activation on the S-scheme heterojunction $Bi_4O_5I_2$/TiO_2 photoanode of a photocatalytic fuel cell for degradation of tetracyclines with green power generation.* Green Energy & Environment, 2026. **11**(3): p. 760-773.
6. Lv, Y., et al., *Visible-light responsive photocatalytic fuel cell based on dual photoelectrodes for efficient wastewater treatment and power generation.* International Journal of Hydrogen Energy, 2026. **198**: p. 152451.
7. Wang, R., et al., *Resourceful treatment of complex uranium-organic wastewater by a hybrid tandem photocatalytic fuel cell with SnS_2 nanoplate modified carbon felt cathode.* Journal of Hazardous Materials, 2024. **480**: p. 135861.
8. Thor, S.-H., et al., *Advanced oxidation treatment of amaranth dye synchronized with electricity generation using carbon-based cathodes in a sustainable photocatalytic fuel cell integrated electro-fenton system.* Journal of Environmental Chemical Engineering, 2021. **9**(6): p. 106439.
9. Zhang, J., et al., *Optimization of rhodamine B degradation and electricity generation in g-C_3N_4/Fe_2O_3/MoS_2/Ti photoanode photocatalytic fuel cell by response surface method.* Journal of Electroanalytical Chemistry, 2025. **987**: p. 119109.
10. Wang, R., et al., *A visible-light-driven hybrid tandem photocatalytic fuel cell system using ZIF-67/CF cathode for enhancing the reduction of U(VI) and oxidation of refractory pollutant and electricity production.* Separation and Purification Technology, 2025. **379**: p. 135080.
11. Vanaja, S., et al., *Multifunctional Z-scheme Bi-MOF/g-C_3N_4 photocatalyst for pharmaceutical degradation, hydrogen evolution, and electricity generation.* Journal of Materials Chemistry A, 2026. **14**(29): p. 18599-18616.
12. Tan, M., et al., *Chromium(VI) removal in a solar-driven photocatalytic fuel cell with carbon quantum dots modified p-type zinc-based metal−organic framework photocathode.* International Journal of Hydrogen Energy, 2023. **48**(49): p. 18719-18730.
13. Liao, Q., et al., *Respective electrode potential characteristics of photocatalytic fuel cell with visible-light responsive photoanode and air-breathing cathode.* International Journal of Hydrogen Energy, 2015. **40**(46): p. 16547-16555.
14. Lam, S.-M., et al., *Z-scheme MoO_3 anchored-hexagonal rod like ZnO/Zn photoanode for effective wastewater treatment, copper reduction accompanied with electricity production in sunlight-powered photocatalytic fuel cell.* Separation and Purification Technology, 2021. **265**: p. 118495.
15. Parvizi, T., J.B. Parsa, and R. Farnood, *Highly-efficient degradation of organic pollutants and synchronous electricity generation in a photocatalytic fuel cell based on the catalytic reactions of hydroxyl and chlorine radicals.* Electrochimica Acta, 2021. **370**: p. 137753.
16. Parvizi, T., J.B. Parsa, and R. Farnood, *Synergetic photocatalytic fuel cell and CuFe layered doubled hydroxide as photoactivator of persulfate for dramatically electricity generation of organic pollutants degradation.* Applied Catalysis B: Environmental, 2022. **319**: p. 121894.
